# The supine position for elbow surgery

**DOI:** 10.1308/003588412X13373405386015n

**Published:** 2012-09

**Authors:** MD Wijeratna, S Thomas, L Van Rensburg

**Affiliations:** Cambridge University Hospitals NHS Foundation Trust,UK

## BACKGROUND

Open elbow surgery is commonly performed through a posterior approach with the patient in the lateral decubitus position.^1^ It is not possible to turn patients who have spinal injuries, unstable pelvic injuries requiring an external fixator and polytrauma patients prior to spinal clearance. We describe a simple technique to overcome these problems while maintaining the access required for emergent and elective elbow surgery.

## TECHNIQUE

**Figure 1:**
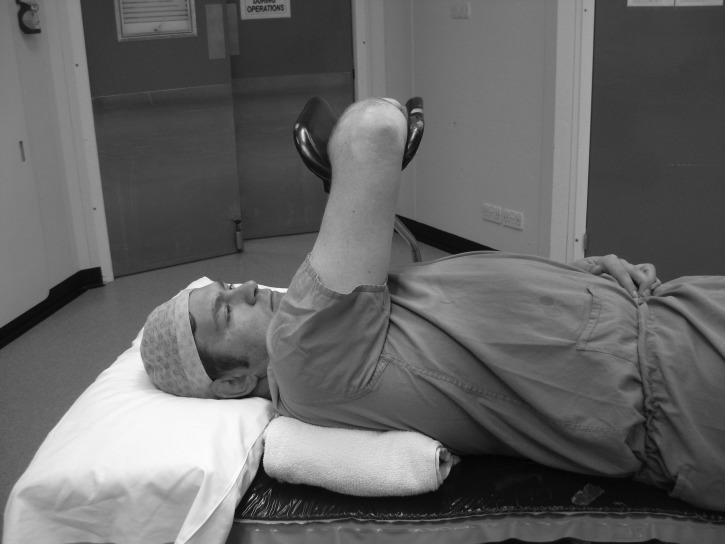
Patient viewed from the side

**Figure 2:**
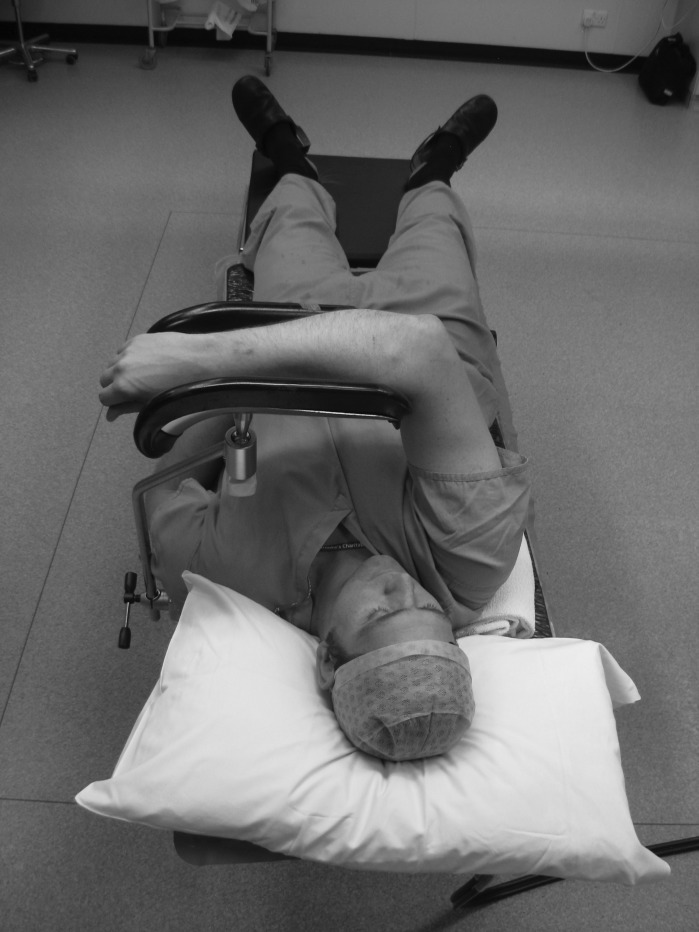
Patient viewed from above

**Figure 3:**
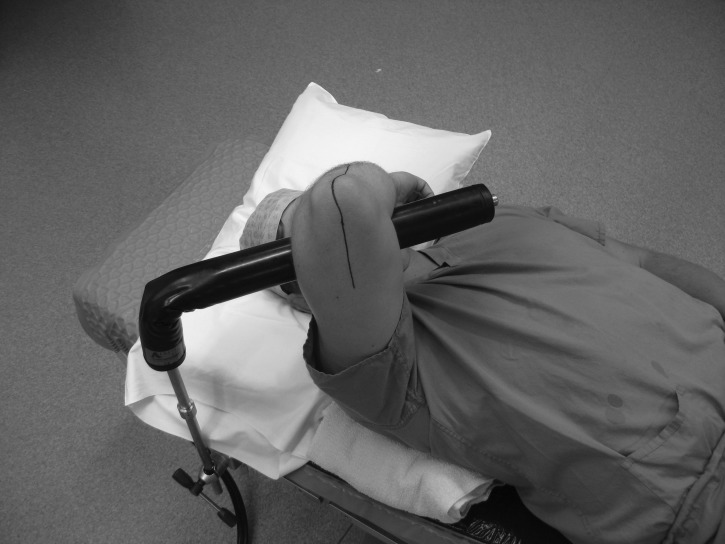
L-bar on ipsilateral side of table

The patient is placed supine and a Lloyd-Davies leg support (commonly used in hip fracture surgery) is attached to the table edge at the level of the contralateral shoulder. The arm to be operated on is supported over the patient’s chest (Figs 1 and 2). A rolled up towel is placed under the ipsilateral shoulder to elevate it and prevent the limb from falling out of the support. If more elbow flexion is required, the Lloyd-Davies leg support can be substituted by a padded L-bar (Fig 3) attached to the ipsilateral table edge. A tourniquet can be applied if required. The C-arm is introduced parallel to the operating table and the elbow extended to obtain fluoroscopic images.

## DISCUSSION

Positioning the patient supine avoids the difficulties of the lateral position. This simple technique for open elbow surgery has been used by the senior author for five years and he has never experienced any limitations of this technique. However, it is not suitable for arthroscopic elbow surgery. The supine position gives excellent visualisation of the distal humerus articular surface and gravity aids the drainage of blood away from the operating field.
